# Assessment of chest CT abnormalities and pulmonary function at 6-month and 1-year after hospital discharge in Chinese patients of COVID-19 pneumonia at the turn of 2022–2023

**DOI:** 10.3389/fmed.2025.1463320

**Published:** 2025-02-26

**Authors:** Xingyu Fang, Jialin Li, Yijun Zhang, Wei Lv, Lin Liu, Yun Feng, Li Liu, Feng Pan, Jinping Zhang

**Affiliations:** ^1^Department of Radiology, The 305 Hospital of People Liberation Army, Beijing, China; ^2^Department of Laboratory, The 305 Hospital of People Liberation Army, Beijing, China; ^3^Department of Health Care, The 305 Hospital of People Liberation Army, Beijing, China

**Keywords:** COVID-19, SARS-CoV-2, pulmonary function, tomography, X-ray, follow-up

## Abstract

**Objective:**

This study aimed to assess chest CT abnormalities and pulmonary function at 6-month and 1-year follow-ups in coronavirus disease 2019 (COVID-19) pneumonia patients of the China epidemic in the turn of 2022–2023.

**Methods:**

A total of 156 hospitalized patients with COVID-19 pneumonia admitted between 29 November 2022 and 10 February 2023 were prospectively assessed at 6-month and 1-year follow-ups. Characteristics and CT scores of pulmonary abnormalities and pulmonary function were compared between different follow-up time points. The correlation of CT abnormalities and pulmonary function at 1-year were evaluated.

**Results:**

Over 1 year, the proportion of pulmonary abnormalities gradually decreased (initial, 100%, 156/156; 6-month, 57.1%, 89/156; and 1-year, 37.8%, 59/156; *P* < 0.001), whereas fibrotic changes increased (initial, 6.4%, 10/156; 6-month, 14.1%, 22/156; and 1-year, 14.7%, 23/56; *P* < 0.001). Compared to participants of the subgroup with nonfibrotic changes, diffusion capacity of the lung for carbon monoxide (DLCO)(*P* = 0.01) and DLCO less than 80% predicted (*P* < 0.001) showed significantly decrease in participants of the subgroup with fibrotic changes. The extent of fibrotic changes was strongly correlated with lower DLCO (*r* = −0.734, *P* < 0.001).

**Conclusion:**

Fibrotic changes might show a tendency to persist over time and correlate strongly with impairment of diffusion function, thus requiring more attention in future follow-ups.

## 1 Introduction

Coronavirus disease 2019 (COVID-19), caused by severe acute respiratory syndrome coronavirus type 2 (SARS-CoV-2), had been shown to cause multiorgan damage, with pulmonary damage being the most common (1, 2). The chest CT abnormalities of COVID-19 pneumonia in the acute phase were predominantly ground-glass opacity (GGO) and consolidation, while residual pulmonary abnormalities mainly include GGO, consolidation, reticulation, linear atelectasis, traction bronchiectasis, and parenchymal bands in the long-term follow-up (3).

Several studies had shown the likelihood of persistent pulmonary fibrotic changes in COVID-19 pneumonia patients with follow-up CT (4–6). Fibrotic changes, interpreted according to the Fleischner Society, commonly refer to the honeycombing sign, traction bronchiectasis, linear atelectasis, and parenchymal bands (7). These changes were more likely to remain long-lasting in the lungs and affected pulmonary function, and thus need more attention. However, the association between residual abnormalities on chest CT and impaired pulmonary function was also inconclusive.

China had experienced a COVID-19 epidemic since gradual deregulation at the end of 2022. Beijing was one of the first and most severe outbreak areas. We prospectively designed a study of participants with COVID-19 pneumonia discharged from hospital in Beijing at the turn of 2022–2023 to investigate the relationship between chest CT residual abnormalities and pulmonary function at 6-month and 1-year follow-up.

## 2 Materials and methods

### 2.1 Participants

This prospective study was approved by the Institutional Review Board of the 305 Hospital of People Liberation Army. Informed consent was provided by all participants.

**FIGURE 1:**
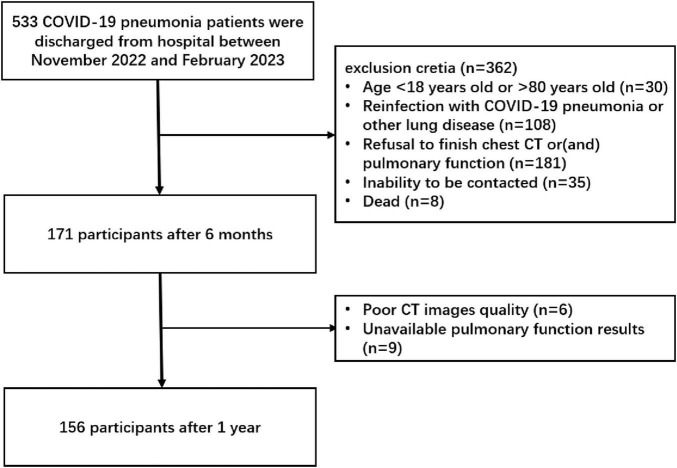
Participant study flowchurt diagram. COVID-19, coronavirus disease 2019.

We prospectively enrolled 156 patients with COVID-19 pneumonia who had been admitted to the 305 Hospital of People Liberation Army between 29 November 2022 and 10 February 2023. All participants presented with symptoms such as fever, sore throat, and cough and were diagnosed of COVID-19 pneumonia by means of a SARS-CoV-2 positive polymerase chain reaction test via nasopharyngeal swabs, then underwent an initial chest CT with consent to confirm the pneumonia. All of these patients completed chest CT and pulmonary function tests at the 6-month and 1-year follow-ups. The exclusion criteria included: age less than 18 years or more than 80 years, reinfection with COVID-19 pneumonia or other lung disease during follow-up period, refusal to be followed up or (and) inability to be contacted, poor CT images quality and unavailable pulmonary function results (patients cannot cooperate well in examinations) (Figure 1).

### 2.2 CT protocol

Non-contrast chest scans were obtained with a 64-section multidetector CT scanner (LightSpeed VCT; GE Medical Systems) or a 128-section multidetector CT scanner (Brilliance iCT; Philips Healthcare), with participants in the supine position during a breath hold following full inspiration. The scanning parameters were 120 kV and adaptive tube current, with the smallest field of view possible according to the body habitus. Axial reconstructions were performed with a section thickness of 1 or 1.25 mm using a bone filter. All 156 patients underwent initial CT scans and follow-ups CT scans using the same parameters.

### 2.3 Image interpretation

All chest CT images were independently assessed by three senior radiologists (Y.Z., Lin.L., W.L., with 19, 18, and 11 years of experience in thoracic radiology, respectively), who were blinded to the baseline and clinical information of the participants. Any disagreement was resolved by discussion and consensus. The readers assessed the CT features using axial images. Multiplane reconstruction was used to resolve any interpretive doubts. Images were interpreted at a window of 1,000–2,000 Hounsfield units and a level of −700 to −500 Hounsfield units, respectively, to assess the lung parenchyma.

CT features were described according to the Fleischner Society glossary (7) as follows: ground-glass opacities (GGO), consolidation, reticulation, linear atelectasis, traction bronchiectasis, parenchymal bands, honeycombing, acute respiratory distress syndrome (ARDS) pattern, crazy paving pattern, organizing pneumonia, and pleural effusion. The CT evidence of pulmonary fibrotic-like changes was defined as presence of linear atelectasis, traction bronchiectasis, parenchymal bands, and honeycombing (6–8).

The chest CT score was calculated per each of the five lung lobes based on the extent of parenchymal involvement (9), as follows: (0) no involvement; (1) <5% involvement; (2) 5%–25% involvement; (3) 26%–50% involvement; (4) 51%–75% involvement; and (5) >75% involvement. The resulting total CT score was the sum of each individual lobar score and ranged from 0 to 25.

### 2.4 Pulmonary function test

The following parameters were measured: forced vital capacity (FVC), forced expiratory capacity at the first second of exhalation (FEV1), total lung capacity (TLC), and diffusion capacity of the lung for carbon monoxide (DLCO) measured by means of the single-breath test. All pulmonary function test measurements were expressed as percentages of predicted normal values. Pulmonary diffusion was regarded as abnormal when the DLCO < 80% of predicted value (10, 11).

### 2.5 Statistical analysis

The statistical analyses were performed using the software SPSS 25.0 (IBM Corp., Armonk, NY, USA). Continuous variables were expressed as medians with interquartile ranges (IQRs) or means ± standard deviations (SDs). Categorical variables were reported as numbers and percentages. Between groups, variables were compared using Chi-squared test (or Fisher’s exact test if any cell frequency ≤5) or independent samples *t*-test, as appropriate. The measurements repeated over time were compared using linear mixed-effects models. Multiple comparisons between two time points were adjusted using the Bonferroni method. Spearman correlation coefficient test was used for the correlation analyses between chest CT scores and with pulmonary function parameters at 1-year follow-up. *P* < 0.05 was considered to indicate a statistically significant difference.

## 3 Results

### 3.1 Participant characteristics

Overall, the study group was composed of 156 participants with a mean age of 62 years ± 14 (SD), and 62 participants were women (39.7%). The baseline and clinical characteristics were summarized in Table 1. Of the 156 participants, the median body mass index was 21.2 kg/m^2^ (IQR, 16.3–29), and 55 (35.3%) were smokers. Ninety-three participants (59.6%) had different types of comorbidities and hypertension (82 participants, 30.3%) was the most common comorbidity. Nine participants (5.8%) had a history of malignant tumor, but none had used pneumotoxic medications in the 3 years prior to infection. The median hospital stay of the participants was 13 days (IQR, 5–22 days), and they underwent 6-month follow-up at a median of 176 days (IQR, 154–203 days) and 1-year follow-up at a median of 363 days (IQR, 341–378 days). Fourteen participants (9.0%) had available chest CT in 1 year prior to infection. One hundred and fifty participants (96.2%) received COVID-19 vaccine before infection, of which 143 (91.7%) received two complete doses of the vaccine and 7 (4.5%) received only one dose. Forty-five participants (28.8%) requiring the highest level of ventilatory support in the form of noninvasive ventilation (32 participants, 20.5%) or invasive positive pressure ventilation (13 participants, 8.3%). Participants were treated with medications mainly including paxlovid (99 participants, 63.5%), azvudine (48 participants, 30.8%), and glucocorticoid (50 participants, 32.1%).

**TABLE 1:** Demographic and clinical characteristics of the participants.

Baseline characteristics	All participants (*n* = 156)
Age, year	62 ± 14
Sex, female	62 (39.7)
Body mass index, kg/m^2^	21.2 (16.3–29)
Smokers	55 (35.3)
Heart rate (beats/min)	89 ± 17
Respiratory rate	21 ± 8
SaO_2_ on room air, %	94 (83–97)
Length of hospital stay, day	13 (5–22)
Time from symptom onset to 6-month follow-up (day)	176 (154–203)
Time from symptom onset to 1-year follow-up (day)	363 (341–378)
Comorbidities	93 (59.6)
Hypertension	54 (34.6)
T2DM	48 (30.8)
IHD	36 (23.1)
COPD	14 (9.0)
Previous VTE	9 (5.8)
Malignant tumor	9 (5.8)
Previous chest CT 1-year before infection	14 (9.0)
Vaccinated patients	150 (96.2)
Ventilatory support	45 (28.8)
Noninvasive ventilation	32 (20.5)
Invasive ventilation	13 (8.3)
Medication	147 (94.2)
Paxlovid	99 (63.5)
Azvudine	48 (30.8)
Glucocorticoid	50 (32.1)

Data are means ± SDs or median (IQR) or *n* (%). SaO_2_, oxygen saturation; IHD, ischemic heart disease; T2DM, type 2 diabetes mellitus; COPD, chronic obstructive pulmonary disease; VTE, venous thromboembolism.

### 3.2 Comparison of CT findings and chest CT scores

**FIGURE 2:**
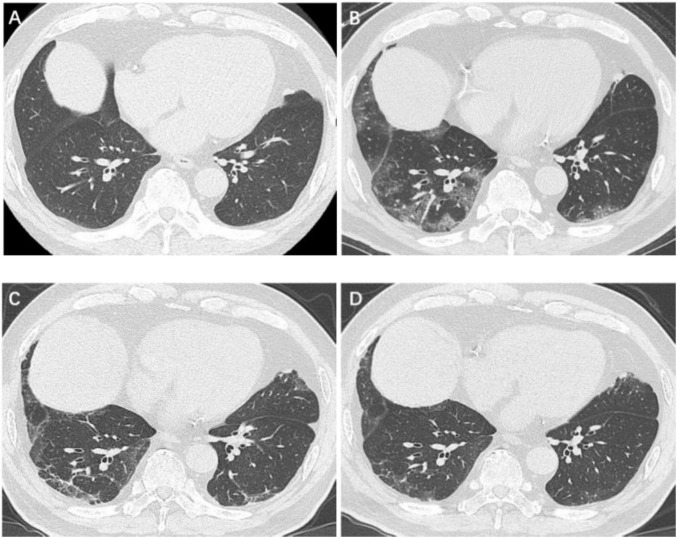
Serial high-resolution noncontrast chest CT in a 67-year-old man with COVID-19 pneumonia. **(A)** Health screening chest CT 5 months prior to COVID-19 infection showed no abnormalities in bilateral lower lobes of lungs. **(B)** Initial CT scans obtained on day 3 after the onset of symptoms showed GGOs in bilateral lower lobes of lungs. **(C)** CT scans obtained on day 188 showed a few GGOs and reticulation bilaterally. **(D)** CT scans obtained on day 361 showed almost absorption of the abnormalities with mild GGOs and reticulation bilaterally. COVID-19, coronavirus disease 2019; GGO, ground-glass opacities.

**FIGURE 3:**
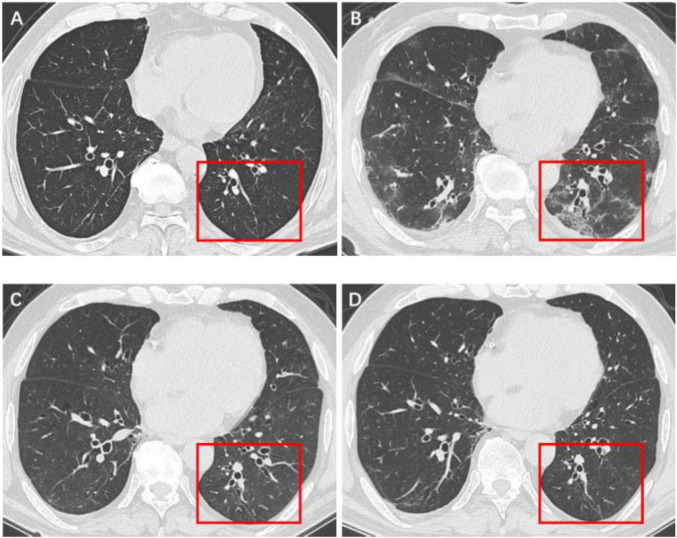
Serial high-resolution noncontrast chest CT in a 61-year-old man with COVID-19 pneumonia. **(A)** Health screening chest CT 3 months prior to COVID-19 infection showed no abnormalities in the lower lobe of left lung (red box). **(B)** Initial CT scans obtained on day 6 after the onset of symptoms showed multiple GGOs and reticulation (red box). **(C)** CT scans obtained on day 173 showed localized residual bronchiolectasis (red box). **(D)** CT scans obtained on day 375 showed persistence of the bronchiolectasis (red box).

Over time, the proportion of participants who showed abnormalities on chest CT gradually decreased (initial, 100%, 156/156; 6-month, 57.1%, 89/156; and 1-year, 37.8%, 59/156; *P* < 0.001) (Table 2). Compared to the initial CT, the proportion of participants with GGO (initial, 100%, 156/156; 6-month, 36.5%, 57/156; and 1-year, 12.8%, 20/156; *P* < 0.001) and consolidation (initial, 49.3%, 77/156; 6-month, 10.3%, 16/156; and 1-year, 4.5%, 7/156; *P* < 0.001) gradually decreased (Figure 2). Meanwhile, participants with reticulation increased from 11 participants (7.0%) to 21 participants (13.5%) at 6-month follow-up and decreased to 18 participants (11.5%) (*P* < 0.001) at 1-year follow-up. Among CT evidence of fibrotic changes, the proportion of participants increased (initial, 6.4%, 10/156; 6-month, 14.1%, 22/156; and 1-year, 14.7%, 23/156; *P* < 0.001) (Figure 3). Compared with the initial CT, chest CT scores of any abnormality, GGO, and consolidation decreased (all *P* < 0.001), whereas fibrotic changes increased (*P* < 0.001) at two follow-up time points. Meanwhile, reticulation showed insignificantly change between three CT scans (*P* = 0.09). Chest CT scores of any abnormality (*P* = 0.02) and GGO (*P* = 0.001) showed significantly decrease between 6-month and 1-year follow-up (Table 3).

**TABLE 2:** Comparison of chest CT findings between initial, 6-month, and 1-year follow-up.

	Initial CT	6-month CT	1-year CT	*P*-value
CT feature				
Any abnormality	156 (100)	89 (57.1)*	59 (37.8)	<0.001
GGO	156 (100)	57 (36.5)*	20 (12.8)	<0.001
Consolidation	77 (49.3)	16 (10.3)*	7 (4.5)	<0.001
Reticulation	11 (7.0)	21 (13.5)	18 (11.5)	<0.001
Fibrotic changes	10 (6.4)	22 (14.1)	23 (14.7)	<0.001
Linear atelectasis	3 (1.9)	6 (3.8)	5 (3.2)	0.02
Traction bronchiectasis	4 (2.6)	9 (5.8)	11 (7.1)	<0.001
Parenchymal bands	4 (2.6)	11 (7.1)	11 (7.1)	<0.001
Honeycombing	3 (1.9)	3 (1.9)	3 (1.9)	0.99
ARDS pattern	3 (1.9)	0 (0)	0 (0)	0.01
Crazy paving pattern	2 (1.3)	0 (0)	0 (0)	0.02
Organizing pneumonia	4 (2.6)	7 (4.5)	5 (3.2)	0.01
Pleural effusion	10 (6.4)	4 (2.6)	5 (3.2)	<0.001

Data are *n* (%).

*Statistically significant compared with 1-year follow-up. GGO, ground-glass opacity; ARDS, acute respiratory distress syndrome.

**TABLE 3:** Comparison of chest CT scores between initial, 6-month, and 1-year.

Chest CT scores	Initial CT	6-month CT	1-year CT	*P*-value
Any abnormality	14 (7–18)	4 (0–9)*	3 (0–7)	<0.001
GGO	11 (7–15)	2 (0–8)*	1 (0–3)	<0.001
Consolidation	4 (1–8)	1 (0–3)	1 (0–3)	<0.001
Reticulation	4 (1–8)	3 (0–7)	3 (0–6)	0.09
Fibrotic changes	0 (0–1)	0 (0–4)	0 (0–5)	<0.001

Data are median (IQR).

*Statistically significant compared with 1-year follow-up. GGO, ground-glass opacity.

### 3.3 Comparison of pulmonary function

Compared with 6-month, the ventilation function indicators of FVC (*P* < 0.001), FEV1 (*P* < 0.001), and TLC (*P* = 0.002) were significantly improved at 1-year follow-up, but there was no significant improvement in FEV1/FVC (*P* = 0.57). Meanwhile, the proportion of patients with FEV1, FVC, and TLC less than 80% predicted were all significantly decreased (all *P* < 0.001) at 1-year follow-up, but there was no significant change in FEV1/FVC < 70% (*P* = 0.09). Compared with 6-month follow-up, DLCO did not improve significantly at 1-year follow-up (*P* = 0.19), but patients with DLCO less than 80% predicted were significantly decreased (*P* < 0.001) (Table 4).

**TABLE 4:** Comparison of pulmonary function between participants at 6-month and 1-year follow-up.

	6-month	1-year	*P*-value
FVC (% predicted)	89 (81–100)	94 (88–101)	<0.001
FEV1 (% predicted)	87 (79–100)	91 (83–101)	<0.001
TLC (% predicted)	86 (80–97)	89 (83–98)	0.002
FEV1/FVC	83 (76–94)	85(77–96)	0.57
DLCO (% predicted)	85 (80–95)	87 (84–96)	0.19
FVC < 80% (% predicted)	37 (24)	17 (11)	<0.001
FEV1 < 80% (% predicted)	41 (26)	30 (19)	<0.001
TLC < 80% (% predicted)	39 (25)	28 (18)	<0.001
FEV1/FVC < 70%	7 (4)	6 (4)	0.09
DLCO < 80% (% predicted)	39 (25)	25 (16)	<0.001

Data are median (IQR) or *n* (%). FVC, forced vital capacity; FEV1, forced expiratory volume in first second; TLC, total lung capacity; DLCO, diffusing capacity of lung for carbon monoxide.

### 3.4 Comparison of pulmonary function and CT findings

At 1-year follow-up, in the subgroup of patients with or without any abnormalities on chest CT, there was no statistically significant difference in all ventilatory function indicators, but the DLCO showed a significant reduction in the subgroup of patients with abnormalities (*P* < 0.001). The proportion of participants with DLCO less than 80% predicted was greater in the subgroup with abnormalities than in the subgroup without abnormalities on chest CT. Similarly, compared to participants of the subgroup with nonfibrotic changes, DLCO (*P* = 0.01) and DLCO less than 80% predicted (*P* < 0.001) showed significantly decrease in participants of the subgroup with fibrotic changes (Table 5).

**TABLE 5:** Comparison of pulmonary function between normal and abnormal CT as well as nonfibrotic changes in CT at 1-year follow-up.

	Normal CT (*n* = 97)	Abnormal CT (*n* = 59)	*P*-value	Nonfibrotic changes (*n* = 36)	Fibrotic changes (*n* = 23)	*P*-value
FVC (% predicted)	95 (90–101)	93 (88–100)	0.41	94 (89–100)	92 (86–100)	0.44
FEV1 (% predicted)	91 (83–101)	90 (83–100)	0.91	90 (83–101)	89 (82–100)	0.87
TLC (% predicted)	91 (84–99)	88 (82–97)	0.09	88 (82–99)	86 (80–97)	0.13
FEV1/FVC	86 (76–97)	84 (75–96)	0.67	85 (75–97)	84 (74–95)	0.91
DLCO (% predicted)	92 (86–99)	84 (78–94)	<0.001	85(80–94)	81 (74–92)	0.01
FVC < 80% (% predicted)	10 (10)	7 (12)	0.42	4 (11)	3 (13)	0.13
FEV1 < 80% (% predicted)	18 (19)	12 (20)	0.99	7 (19)	5 (22)	0.34
TLC < 80% (% predicted)	17 (18)	11 (19)	0.59	7 (19)	4 (17)	0.59
FEV1/FVC < 70%	4 (4)	2 (3)	0.11	1 (3)	1 (4)	0.07
DLCO < 80% (% predicted)	8 (8)	17 (29)	<0.001	9 (25)	8 (35)	<0.001

Data are median (IQR) or *n* (%). GGO, ground-glass opacity; FVC, forced vital capacity; FEV1, forced expiratory volume in first second; TLC, total lung capacity; DLCO, diffusing capacity of lung for carbon monoxide.

### 3.5 Correlation between pulmonary function and chest CT scores

The extent of fibrotic changes was strongly correlated with lower DLCO (*r* = −0.734, *P* < 0.001) while the extent of reticulation was moderately correlated with lower DLCO (*r* = −0.661, *P* < 0.001). GGO extent moderately correlated with lower FVC (*r* = −0.577, *P* = 0.001), as was the extent of consolidation (*r* = −0.598, *P* = 0.001). There was no more significant correlation between the other pulmonary function indicators and chest CT scores of abnormalities (Table 6).

**TABLE 6:** Correlation of chest CT scores with pulmonary function at 1-year follow-up.

	FVC (% predicted)	FEV1 (% predicted)	TLC (% predicted)	DLCO (% predicted)
GGO	*r* = −0.577 *P* = 0.001	*r* = −0.499 *P* = 0.006	*r* = −0.339 *P* = 0.072	*r* = −0.380 *P* = 0.042
Consolidation	*r* = −0.598 *P* = 0.001	*r* = −0.463 *P* = 0.011	*r* = −0.488 *P* = 0.007	*r* = −0.281 *P* = 0.139
Reticulation	*r* = −0.194 *P* = 0.313	*r* = −0.263 *P* = 0.168	*r* = −0.425 *P* = 0.021	*r* = −0.661 *P* < 0.001
Fibrotic changes	*r* = −0.245 *P* = 0.200	*r* = −0.134 *P* = 0.488	*r* = −0.254 *P* = 0.183	*r* = −0.734 *P* < 0.001

A Spearman correlation coefficient value (*r*) < 0.4, between 0.4 and 0.69, between 0.7 and 0.9, and exceeding 0.9 represented poor, moderate, strong, and very strong correlations, respectively. FVC, forced vital capacity; FEV1, forced expiratory volume in first second; TLC, total lung capacity; DLCO, diffusing capacity of lung for carbon monoxide.

## 4 Discussion

Long-term effects of COVID-19 pneumonia on the lungs remained inconclusive. Currently, several chest CT-based studies had found that COVID-19 pneumonitis retains prevalence residual pulmonary abnormalities, and the overall proportion of chest CT abnormalities varied widely (7.1%–96.7%) at 1-year follow-up (12). The reasons for the variation might be related to differences in the characterization of viral subtypes due to continuous virus mutation, as well as the selection criteria and size of the samples in the different studies. In our study, 37.8% (55/156) of patients still had pulmonary abnormalities at 1-year follow-up, a significant decrease relative to 6-month follow-up (57.1%, 89/156). Of these, GGO was the most pronounced reduced, but it was still the highest percentage (12.8%, 20/156) of pulmonary abnormalities on chest CT at 1-year follow-up, which was compatible with a several previous studies (13, 14). GGOs represented alterations of different pathologies, such as intra-alveolar exudates or thickening of the intrapulmonary interstitium, and long-existing GGO may tend to the latter. A previous study using photon-counting detector CT found that a considerable proportion of long-existing subpleural GGOs contained bronchiectasis (15).

Several studies had reported pulmonary fibrotic changes on chest CT in COVID-19 pneumonia patients at 1-year follow-up (6, 14, 16). Lee et al. (17) concluded in a meta-analysis that fibrotic-like abnormalities persisted through multiple follow-ups during 1-year period. In a study, Kumar et al. (18) found post-COVID-19 chest CT features of irreversible pulmonary fibrosis remain static over time. This was consistent with our findings of fibrotic changes (6-month, 14.1%, 22/156; 1-year, 14.7%, 23/156) and chest CT scores of fibrotic changes [6-month, 0, IQR (0–4) vs. 1-year, 0, IQR (0–5)]. Unchanging fibrotic changes were important precursors to idiopathic fibrosis (19, 20). Therefore, long-standing fibrotic abnormalities required ongoing attention. According to previous experience, a long-term follow-up found that some fibrotic abnormalities persisted 14 years in SARS-CoV-1 patients after infection (21).

The correlation between residual CT abnormalities and pulmonary function in COVID-19 pneumonia patients was inconclusive. Compared to abnormalities on chest CT, pulmonary function tests could more directly and accurately reflect the extent of pulmonary impairment and were therefore of greater concern (22, 23). Our study found that impairment of diffusion function was significant at 1-year follow-up in patients with fibrotic changes relative to patients without fibrotic changes. Han et al. (24) also found in a 2-year follow-up study of COVID-19 pneumonia that patients with pulmonary fibrotic changes had more diffusion function impairment. Of note, our study found that some patients’ chest CT recovered to normal at 1-year follow-up, but mild impairment of diffuse function remained (8%, 8/97). This might be related to the limitations of conventional chest CT. Several studies had shown that abnormalities could also be detected by other methods (e.g., expiratory chest CT) in patients with COVID-19 pneumonia who had normal conventional chest CT (25, 26).

In our study, reticulation was found to have a moderate correlation with impairment of diffusion function (*r* = −0.661, *P* < 0.001). Previous study found that reticular abnormalities might also indicate fine interstitial fibrosis, which was irreversible in chronic interstitial lung disease (27). In some previous studies of COVID-19 pneumonia, reticulation was also found to persist during follow-ups (6, 24). Hence, it was necessary to pay closer attention to and better understand the development of reticular patterns after COVID-19 pneumonia.

Our study had some limitations. Firstly, during the infectious phase of COVID-19, pulmonary function tests were strictly limited to avoid disease transmission. As a result, we were lacking data from the initial pulmonary function tests, which had an impact on the overall comparison of the data. Secondly, no histopathologic evaluation was performed to corroborate the CT findings. Fibrotic changes were defined purely radiologically, based on associated CT abnormalities.

## 5 Conclusion

This prospective study showed that in the COVID-19 epidemic of China during the turn of 2022–2023, 6-month and 1-year follow-ups showed a gradual decrease in overall pulmonary abnormalities and CT scores. However, fibrotic changes might show a tendency to persist over time and correlate strongly with impairment of diffusion function, and therefore changes of these features should be paid more attention to in future follow-ups.

## Data Availability

The raw data supporting the conclusions of this article will be made available by the authors, without undue reservation.
